# Supplementary figures and images for: Girdin is a component of the lateral polarity protein network restricting cell dissemination
Source: PLoS Genet. 2020 Mar 20;16(3):e1008674. doi: 10.1371/journal.pgen.1008674 (PMC7112241; doi:10.1371/journal.pgen.1008674)

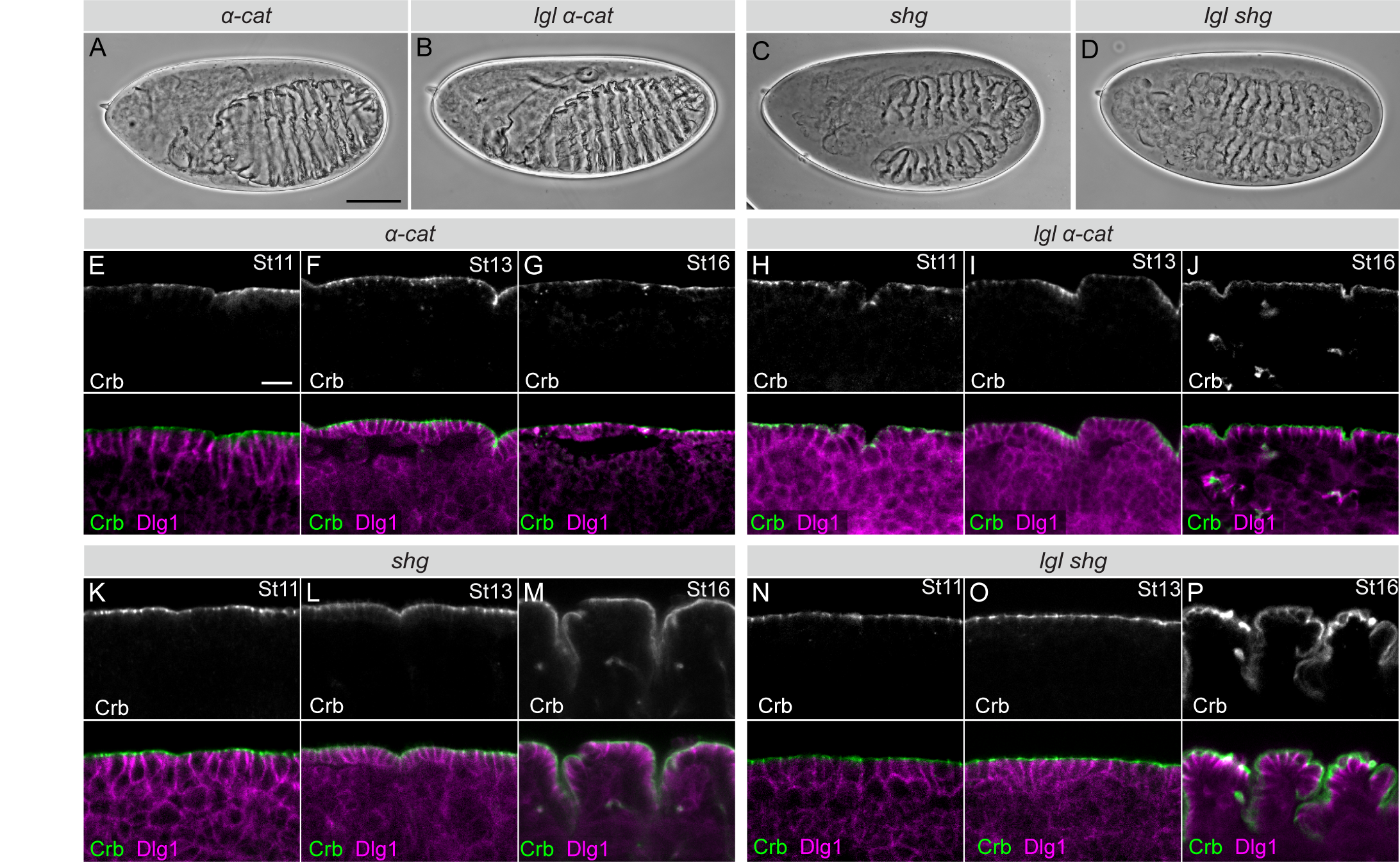

Supplement: S1 Fig — A-D, Cuticle preparations of whole mounted embryos of the indicated genotypes. Scale bar in A represents 100 μm, and also applies to B-D. E-P, Embryos at embryonic stage (St) 11, 13 or 16 were fixed and processed for immunofluorescence. The distribution of the apical marker Crb and of the lateral protein Dlg1 was then assessed by confocal microscopy in the ventral ectoderm or the ventral epidermis. Scale bar in E represents 10 μm, and also applies to F-P. A-P show representative results of experiments that were performed in triplicate. At least 20 embryos were analyzed in each replicate. (TIF) [file pgen.1008674.s001.tif]

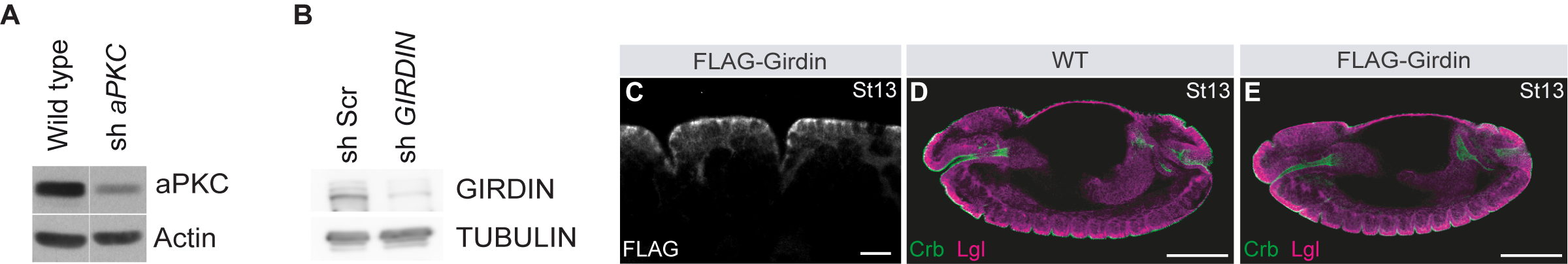

Supplement: S2 Fig — A-B, Western blots showing knockdown efficiency for aPKC (A), and GIRDIN (B). Actin (A) or TUBULIN (B) were used as loading control. C, Embryos expressing FLAG-Girdin were fixed and immunostained with anti-FLAG antibodies. D-E, Crb (green) and Lgl (magenta) distribution in a wild type embryo (D) or a FLAG-Girdin expressing specimen (E). Panels depict whole embryo view (anterior is to the left, and dorsal is up). Scale bar in C = 10 μm, scale bar in D, E = 100 μm. (TIF) [file pgen.1008674.s002.tif]

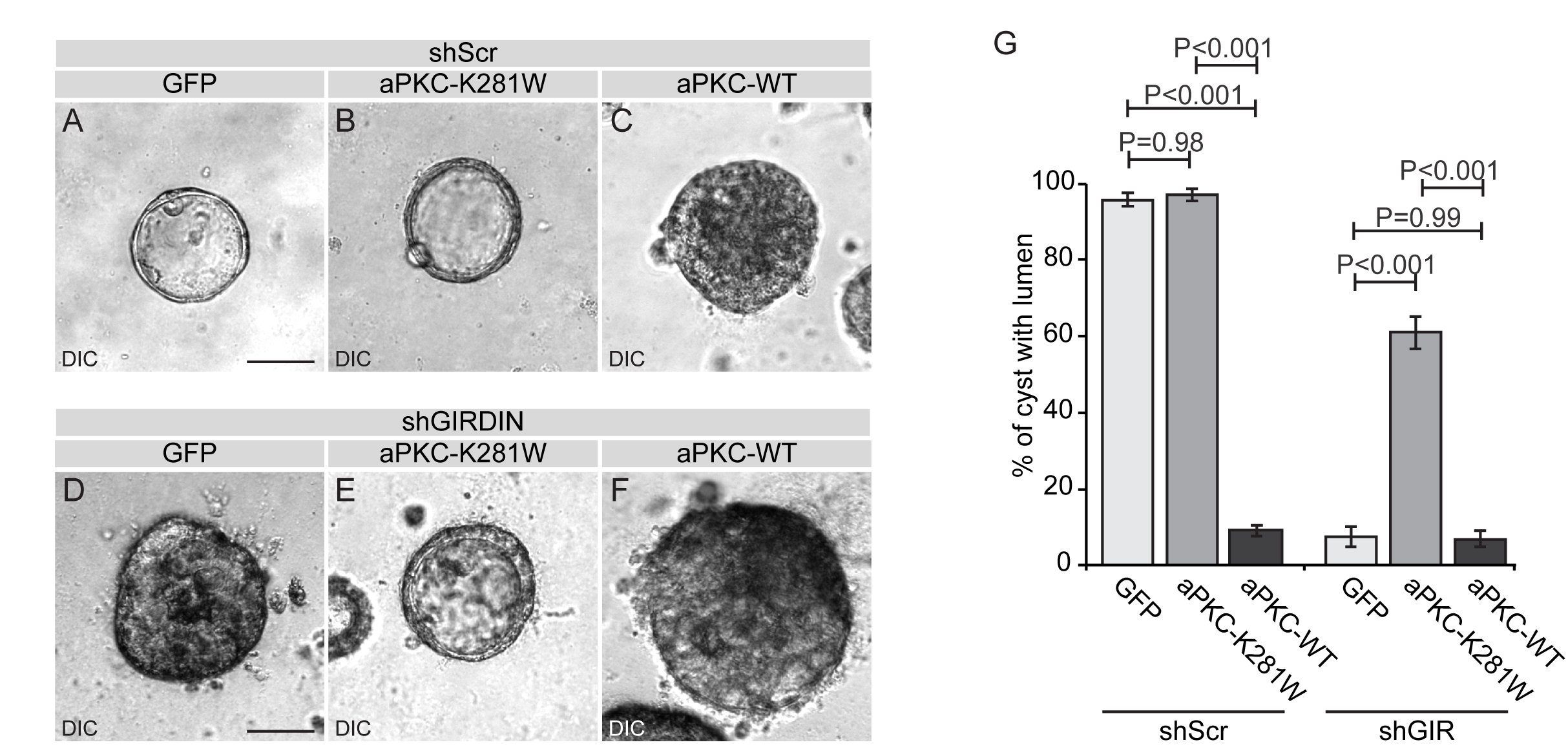

Supplement: S3 Fig — Kinase-deficient aPKC restores lumen formation in GIRDIN-deficient cells. A-F, Caco-2 cell cysts after 7-days in culture were visualized by DIC microscopy. GIRDIN-deficient (shGIR) or control cells (shScr) expressed GFP (control), wild-type (aPKC-WT), or kinase-deficient (aPKC-K281W) aPKC. Scale bars represent 50 μm. G, Histogram displaying the proportion of 3D cellular structures with a single prominent lumen (shScr/GFP, n = 486; shScr/aPKC-K281W, n = 309; shScr/aPKCWT, n = 341; shGIR/GFP, n = 292; shGIR/aPKC-K281W, n = 229; shGIR/aPKC-WT, n = 227; r = 3 independent experiments). Error bars = sd. Differences were determined using ANOVA with Tukey HSD. (TIF) [file pgen.1008674.s003.tif]

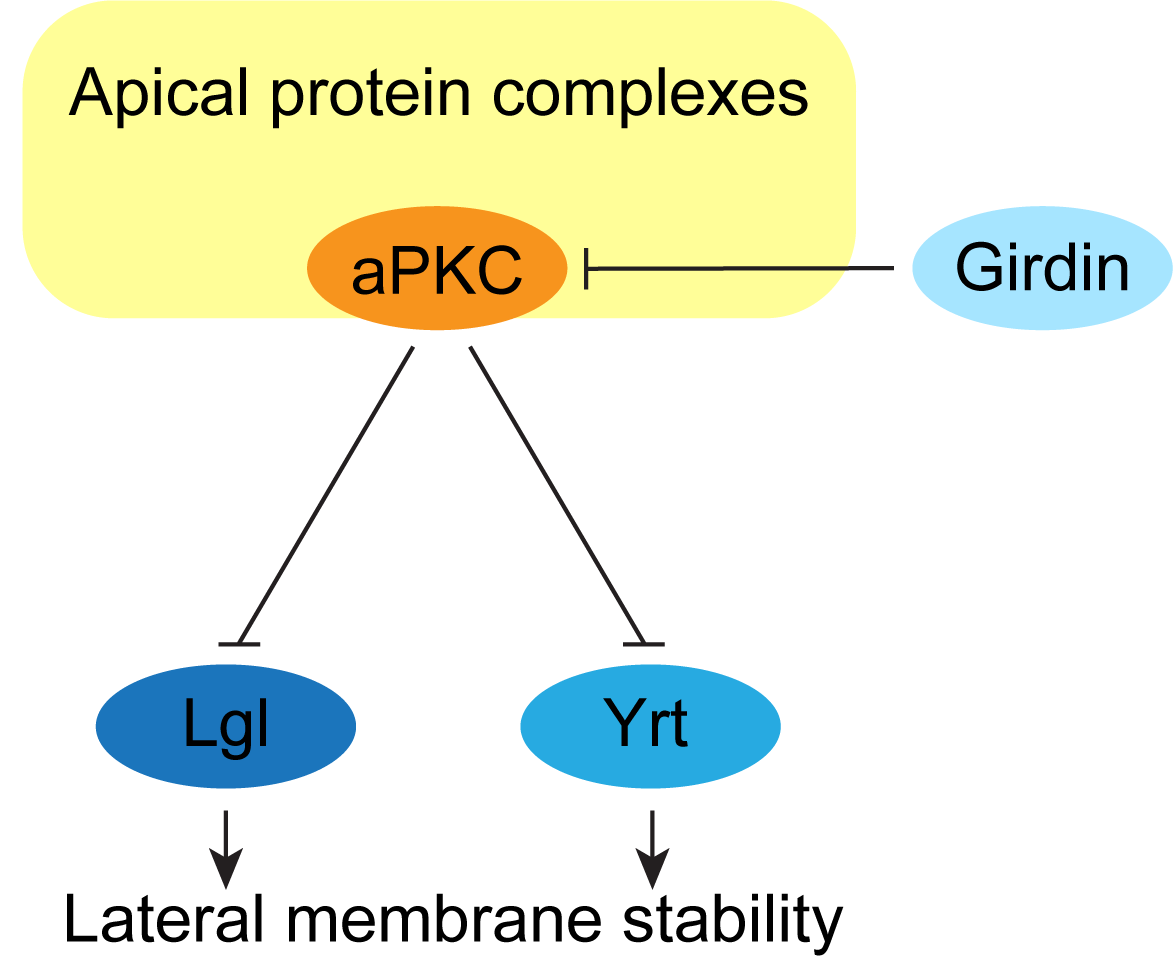

Supplement: S4 Fig — Our data indicate that Girdin cooperates with Lgl and Yrt. The latter two proteins act in parallel pathways to antagonize the apical machinery, thereby supporting lateral membrane stability [8,27,28,30]. Although Lgl and Yrt act independently, they have in common that they are both negatively regulated by aPKC [15,24,25]. We provide evidence that Girdin antagonizes aPKC function. We thus propose a model in which Girdin supports the function of Yrt and Lgl by restricting the activity of aPKC. (TIF) [file pgen.1008674.s004.tif]
